# Utilizing Consumer Technology (Apple’s ResearchKit) for Medical Studies by Patients and Researchers: Proof of Concept of the Novel Platform REach

**DOI:** 10.2196/jopm.9335

**Published:** 2018-04-04

**Authors:** Marleen MHJ van Gelder, Lucien JLPG Engelen, Thijs Sondag, Tom H van de Belt

**Affiliations:** ^1^ Radboud REshape Innovation Center Radboud University Medical Center Nijmegen Netherlands; ^2^ Department for Health Evidence Radboud Institute for Health Sciences Radboud University Medical Center Nijmegen Netherlands; ^3^ Process Improvement & Health Care Innovations Radboud University Medical Center Nijmegen Netherlands

**Keywords:** data collection, HealthKit, platform, PRIDE Study, smartphone, wearables

## Abstract

Medical research suffers from declining response rates, hampering the quest for answers to clinically relevant research questions. Furthermore, objective data on a number of important study variables, such as physical activity, sleep, and nutrition, are difficult to collect with the traditional methods of data collection. Reassuringly, current technological developments could overcome these limitations. In addition, they may enable research being established by patients themselves provided that they have access to a user-friendly platform. Using the features of Apple's ResearchKit, an informed consent procedure, questionnaire, linkage with HealthKit data, and “active tasks” may be administered through a publicly available app. However, ResearchKit requires programming skills, which many patients and researchers lack. Therefore, we developed a platform (REach) with drag and drop functionalities producing a ready-to-use code that can be embedded in existing or new apps. Participants in the pilot study were very satisfied with data collection through REach and measurement error was minimal. In the era of declining participation rates in observational studies and patient involvement, new methods of data collection, such as REach, are essential to ensure that clinically relevant research questions are validly answered. Due to linkage with HealthKit and active tasks, objective health data that are impossible to collect with the traditional methods of data collection can easily be collected.

## Introduction

The growing number of smartphones in both the developed and developing world (2014: 2.6 billion; 2020: 5.9 billion) and the rapidly expanding coverage of Long-Term Evolution (4G) networks [1], combined with numerous wearable devices such as activity trackers and smart watches, provide unique possibilities to reach potential study participants worldwide on a device they use multiple hours per day [2]. In addition, current technological developments may enable research being established by patients themselves provided that they have access to a user-friendly platform. For example, in the Health app that is available on iPhones since 2014, health and fitness data collected in other apps and wearable devices are put together in one place through HealthKit. Consequently, Apple introduced ResearchKit in March 2015, an open source framework that enables researchers to create iOS apps for medical research [3]. The first studies using ResearchKit for data collection have recently been published [4-7].

The core ResearchKit framework consists of 3 modules handling informed consent, surveys, and “active tasks”. For the latter, data are collected through the iPhone sensors including the accelerometer, gyroscope, Multi-Touch display, and microphone. However, although ResearchKit claims to be an easy-to-use platform for researchers to create research apps, involvement of a developer may in fact be necessary to incorporate all needs. For example, iOS and Swift programming skills are necessary to develop an app for a medical study using ResearchKit. Therefore, we developed REach, a platform that enables both patients and researchers to collect data through an app using the main features of ResearchKit. Its reliability and usability were assessed in a pilot study among postpartum women.

## How the Innovation Works

**Figure 1:**
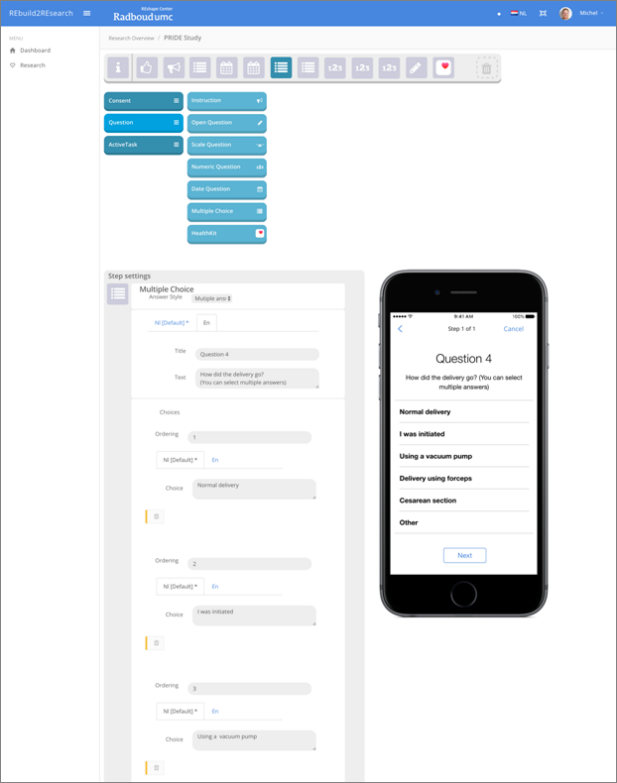
Overview of the REach Web app.

REach was developed by the Radboud REshape Innovation Center in cooperation with patients and researchers from various medical disciplines, including epidemiology, health technology assessment, pediatrics, and medical informatics. It consists of two sections: a Web app in which the investigator (patient or researcher) can set up the study, and an app available in the App Store with which data are collected from participants. Using drag and drop, an informed consent procedure, questions, and active tasks may be easily added to a study in the Web app (Figure 1).

A full informed consent procedure, consisting of displaying the consent documents, participant name entry, and the participant’s signature, is available in REach. The core of ResearchKit has already received numerous endorsements as a secure platform because the data are stored highly encrypted, only on the smartphone itself [8]. It is considered one of the most, if not the most, secure platforms available at present. Once the participant signs the informed consent form, the document is available for the investigator as a PDF file for archiving purposes.

Within the Web app, the investigator may build a regular questionnaire with instructions, multiple choice questions, open-ended questions (literal or numerical), rating scales, and date/time questions. Comparable to Web-based questionnaires, validity checks may be included to improve data quality. In addition, HealthKit questions may be added, in which the participant is asked for consent to share already collected data on for example body weight, heart rate, and steps. This enables investigators to easily collect unique and objective data on the health of study participants.

In addition to completing the questionnaire, data may be collected from the participants by having them performing active tasks. A number of active tasks are predefined in ResearchKit, which enable inviting participants to perform activities under partially controlled conditions using iPhone sensors for data collection. These active tasks fall into 6 categories: motor activities, fitness, cognition, voice, audio, and hole peg [9]. Currently, two active tasks are available within REach. With tone audiometry, the minimum amplitude for the participant to recognize the sound is determined. Reaction time uses the smartphone’s accelerometer and gyroscope to collect data on device motion.

Once the study has been set up in the Web app, the investigator may start data collection. Studies in the app can be open to the public or by invitation only. For the latter, the email address of the potential participant should be available. The app may be free or paid, enabling possibilities for crowdfunding. There are no restrictions on the geographical location or number of study participants, but an individual can only participate once in a certain study. Furthermore, the app is available in multiple languages, depending on the language settings of the iPhone (default: English). In the dashboard, the investigator may monitor the status of the study (number of views, number of completed consent forms, and number of completed participations). The resulting data file can be downloaded at any time during or after completion of data collection and imported in statistical software packages.

## Pilot Testing

### Methods

Data collection through REach was pilot tested in a subsample of participants in the PRegnancy and Infant DEvelopment (PRIDE) Study [10]. All women who delivered a live-born singleton and completed the PRIDE Study’s first postpartum Web-based questionnaire (2 months after the estimated date of delivery) between October 2015 and March 2016 were invited to test REach (n=463). The app was developed by a researcher without needing to consult the developer. Testing of the app involved providing informed consent, completing 10 questions comparable to those in the Web-based questionnaire to get more insight into its reliability, and possible sharing of HealthKit data. The active tasks were not used in this population.

Using REach, we collected data on the occurrence of pregnancy complications (“check all that apply” format), estimated date of delivery and actual date of delivery (to calculate gestational age), birth weight, birth length, head circumference, birth defects, and closed-ended questions on mode of delivery, presentation, and infant sex. The questions in the Web-based questionnaire on perinatal outcome, including birth weight (intraclass correlation coefficient [ICC] 0.96) and birth length (ICC 0.90), were previously validated [11].

Furthermore, participants were asked to complete a short evaluation questionnaire, which included the System Usability Scale [12]. The app and evaluation questionnaire were administered at least 2 weeks after the postpartum Web-based questionnaire. To assess reliability, we calculated kappa statistics and ICCs with 95% confidence intervals for categorical and continuous variables, respectively. IBM SPSS Statistics for Windows, Version 22 (IBM Corp, Armonk, NY) was used for all statistical analyses.

### Results

A total of 31 women tested the app. The results of the reliability analyses for the categorical variables are shown in Table 1. In general, there were very few discrepancies between the data collected through the app and through the Web-based questionnaire for pregnancy complications, mode of delivery, presentation at birth, and infant sex. The relatively high numbers of false negatives for nausea and vomiting of pregnancy and extreme fatigue mainly included women who reported these complication in the baseline questionnaire, which is administered at the end of the first trimester. Therefore, the effect of time may play a bigger role than underreporting of these two complications in the app itself.

**Figure 2:**
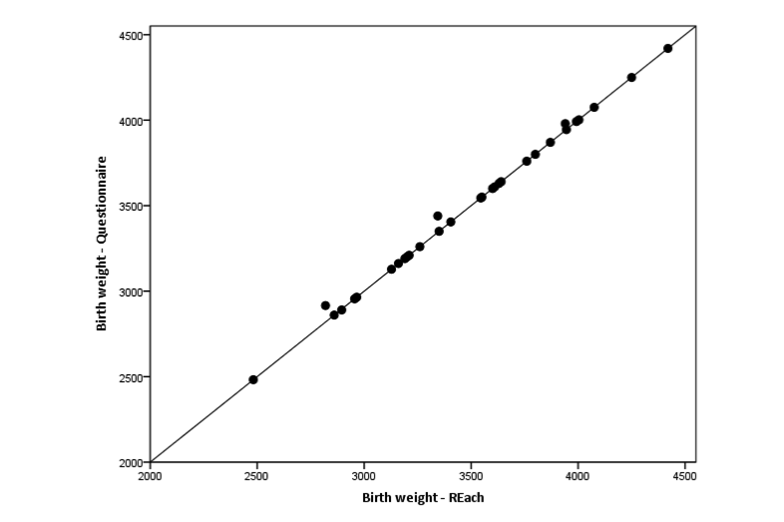
Comparison of birth weight (in grams) reported by mothers in the REach application and in the Web-based questionnaire (N=31).

**Figure 3:**
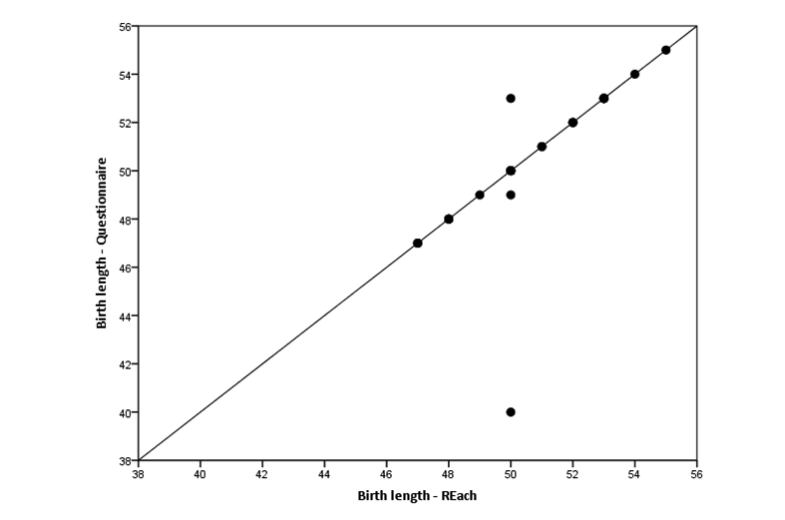
Comparison of birth length (in cm) reported by mothers in the REach application and in the Web-based questionnaire (N=29).

One woman reported diagnosis of a birth defect in both the app and the questionnaire. Agreement between the questionnaire and app was excellent for birth weight (ICC 1.00, 95% CI 1.00-1.00; Figure 2), but substantially lower for birth length (ICC 0.73, 95% CI 0.50-0.86; Figure 3). However, this outlier seemed to be caused by the respondent making a typo in the questionnaire. Omitting this subject from the analysis increased the ICC to 0.97 (95% CI 0.93-0.98).

HealthKit data were shared by 11 of the 31 participants (35%); only the number of steps per day was shared. For the remaining 20 participants, we cannot distinguish between those who granted sharing of data but had no data available, and participants who denied permission to share data. Although insight into this matter would be interesting from a research perspective, HealthKit does not allow sharing of these data to avoid information leaks and to protect user privacy.

We received 25 evaluation questionnaires. The participants did not report problems using the app. The mean score on the System Usability Scale was 83.9 (SD 10.7), indicating a nearly excellent level of satisfaction [13]. On a scale from 1 (worst) to 10 (best), the mean rating was 7.8 (0.7). Only 2 participants (8%) preferred a Web-based questionnaire to completing the questions through the app; the majority either preferred the app (56%) or had no preference (36%).

**Table 1:** Comparison of app and questionnaire data for categorical variables. N/A: not applicable.

Variable		App positive		App negative		Kappa statistic
		Questionnaire positive	Questionnaire negative	Questionnaire positive	Questionnaire negative	
**Pregnancy complications**						
	Nausea and vomiting of pregnancy	6	0	5	20	0.68
	Extreme fatigue	3	0	16	12	0.13
	Gestational hypertension	1	1	0	29	0.65
	Preeclampsia	0	0	0	31	N/A
	Gestational diabetes	0	0	0	31	N/A
	Thyroid disorders	0	0	0	31	N/A
	Pelvic girdle pain	5	1	1	24	0.79
	Anemia	1	0	1	29	0.65
**Mode of delivery**						
	Unassisted vaginal delivery	24	0	0	7	1.00
	Cesarean section	7	0	0	24	1.00
Breech presentation		2	0	0	31	1.00
Male infant		12	0	0	18	1.00

## Conclusion

In the era of declining participation rates in observational studies and patient involvement [14,15], adding new methods of data collection to the toolbox of medical researchers, such as REach, is essential to ensure that clinically relevant research questions validly answered. Despite some methodological limitations of the pilot study, including the relatively small sample size and selective participation as only iPhone users could be included, study participants were very satisfied with data collection through smartphones and measurement error seemed minimal. Although no formal validation analyses were conducted in the few other studies in which ResearchKit was used for data collection, data quality was also reported to be high and consistent [5-7].

Due to the linkage with HealthKit and the incorporation of active tasks, objective health data that are impossible to collect with the traditional methods of data collection can easily be collected. However, HealthKit data will probably not be available for the complete study population due to declining to share this information or not using the HealthKit on the iPhone at all, yielding the possibility for selection bias.

REach is currently available through the website of the Radboud REshape Innovation Center [16]. More extensive tests of the platform, including patient-initiated studies, are ongoing and possibilities for platforms for other mobile operating systems, such as Android, are now being explored.

## Abbreviations

PRIDE Study: PRegnancy and Infant DEvelopment Study
ICC: intraclass correlation coefficient
